# Development and validation of a tool to measure collaborative practice between community pharmacists and physicians from the perspective of community pharmacists: the professional collaborative practice tool

**DOI:** 10.1186/s12913-022-08027-w

**Published:** 2022-05-14

**Authors:** Ana I. Sanchez-Molina, Shalom I. Benrimoj, Ramon Ferri-Garcia, Fernando Martinez-Martinez, Miguel Angel Gastelurrutia, Victoria Garcia-Cardenas

**Affiliations:** 1 Pharmaceutical Care Research Group, University of Granada, Granada, Spain; 2 Department of Statistics and Operations Research, University of Granada, Granada, Spain; 3 Graduate School of Health, University of Technology, Sydney, Australia

**Keywords:** Community pharmacists, Physicians, Collaborative practice, Models, Interprofessional collaboration, Tool, Measurement

## Abstract

**Background:**

Collaborative practice between community pharmacists and physicians is becoming increasingly common. Although tools and models to explore collaborative practice between both health care professionals have been developed, very few have been validated for their use in clinical practice. The objective of this study was to develop and validate a tool for measuring collaborative practice between community pharmacists and physicians from the perspective of community pharmacists.

**Methods:**

The DeVellis method was used to develop and validate the Professional Collaborative Practice Tool. A pool of 40 items with Likert frequency scales was generated based on previous literature and expert opinion. This study was undertaken in Spain. A sample of community pharmacists providing medication reviews with follow-up and a random sample of pharmacists providing usual care were invited to participate. Exploratory and confirmatory factor analysis was used to assess the tool’s reliability and content validity.

**Results:**

Three hundred thirty-six pharmacists were invited with an overall response rate of 84.8%. The initial 40 items selected were reduced to 14 items. Exploratory Factor Analysis provided a 3-factor solution explaining 62% of the variance. Confirmatory Factor Analysis confirmed the three factors “Activation for collaborative professional practice,” the “Integration in collaborative professional practice,” and the “Professional acceptance in collaborative professional practice.” The tool demonstrated an adequate fit (X^2^/df = 1.657, GFI = 0.889 and RMSEA = 0.069) and good internal consistency (Cronbach’s alpha = 0.924).

**Conclusions:**

The Professional Collaborative Practice Tool has shown good internal reliability and criterion validity. The tool could be used to measure the perceived level of collaborative practice between community pharmacists and physicians and monitor changes over time. Its applicability and transferability to other settings should be evaluated.

**Supplementary Information:**

The online version contains supplementary material available at 10.1186/s12913-022-08027-w.

## Introduction

Considering the global challenges of an aging population, the increase in chronic conditions, and the increased demand for health services, there is a need to provide multidisciplinary and coordinated care to patients. According to the World Health Organization, *“collaborative practice in health-care occurs when multiple health workers from different professional backgrounds provide comprehensive services by working with patients, their families, careers, and communities to deliver the highest quality of care across settings”* [1]. Improved collaboration between healthcare providers has been suggested as a strategy to optimize health care systems [2]. Patients often require multiple healthcare professionals, and whilst this inter-professional care may create a more complex healthcare model, there is evidence it increases efficiency [3, 4] and consumer safety [5, 6]. It also positively influences the job satisfaction of healthcare professionals [7], improves the continuity in the coordination of care, and reduces health care costs [8, 9].

Collaborative practice between community pharmacists and physicians (i.e., primary care physicians) is becoming increasingly common [10–12]. In many countries, the community pharmacists’s role has traditionally been focused on dispensing medicines. However, this role is now evolving towards providing direct patient care [13]. The provision of professional pharmacy services embodies this patient-directed philosophy aiming at improving the quality use of medicines and ultimately patient outcomes [14]. They range from basic activities like providing medicines information, to more complex clinical decision-making services such as comprehensive medication reviews or disease state management, which often involve an increased collaboration with physicians [15]. While community pharmacists and physicians have traditionally worked in isolation from each other, several studies have established that increased collaboration between both health professionals can improve medication management, leading to positive patient health outcomes [16, 17]. Several studies have explored the collaboration between pharmacists and physicians, mainly focusing on barriers and facilitators [18] and on the development of theoretical approaches to explain the nature of the collaboration.

In 2015, Bardet et al. published a systematic review of collaboration models between community pharmacists and physicians [19] and identified four models of collaboration: The Collaborative Working Relationship Model (CWR) [20], the Conceptual Model of GP-Community Pharmacists Collaboration [21], the Community Pharmacist Attitudes towards Collaboration with GPs Model (ATC-P) [22], and the GP Attitudes Towards Collaboration with Community Pharmacists (ATC-GP) [23]. Furthermore, this review proposed a framework for collaboration: the Physician-Community Pharmacy Collaboration Meta-Model. This framework is based on the analysis of the four models cited above and summarized the key elements for collaboration: trust, interdependence, perceptions and expectations about the other health care professional, skills, interest for collaborative practice, role definition and communication.

The CWR [20] is a widely cited collaborative model between community pharmacists and physicians. This framework was based on models of interpersonal relationships (including theories of social exchanges and business relationships), and collaborative care models, primarily relating to nurses and physicians. The model hypothesizes that the development of working relationships between pharmacists and physicians evolves through several phases, advancing from brief interactions to mutually beneficial partnerships with defined roles and patient-care responsibilities. However, these theoretical phases have never been validated for their use in practice. Zillich et al. developed the “Physician–Pharmacist Collaboration Instrument” (PPCI) [24], predominantly based on the CWR model, which was tested from the perspective of the physician but with a small sample size. The “Conceptual Model of GP-Pharmacist Collaboration,” was derived from interviews with physicians and community pharmacists in the United Kingdom. Similarly, this model appears not to have been tested or validated in practice from the perspective of the pharmacist [21]. Both models, the Community Pharmacist Attitudes Towards Collaboration with GPs Model (ATC-P) and the GP Attitudes Towards Collaboration with Community Pharmacist (ATC-GP) describe factors that describe community pharmacists and physician attitudes to collaborate. The (ATC-P) [22] and (ATC-GP) [23] are two empirical models which were developed based on the experience after implementation home medication review and diabetes medication assistance services in Australia.

In Spain, physicians are employees of the national health system and work with other health care professionals in primary health care centers, whilst community pharmacists are considered to be external contractors to the system. This context is similar to other countries, where community pharmacies are not co-located with medical practitioners and are contracted by governments to dispense medications. However, the concept of collaborative practice between community pharmacists and physicians is gaining importance, due to an increased provision of professional pharmacy services such as Medication Review with Follow-up (MRF) [25]. According to the Pharmaceutical Care Network Europe, MRF is a type 2 medication management review, with the objective of detecting and resolving drug related problems to optimise therapeutic outcomes. This service requires the close collaboration between the two professionals to ensure optimal patient medication management [26, 27]. Although tools and models to explore collaborative practice have been proposed for their use in Spain, these are yet to be validated for their use in clinical practice. The objective of this study was to develop and validate a tool to measure collaborative practice between physicians and community pharmacists from the perspective of community pharmacists.

## Methods

The eight-step method proposed by DeVellis et al. [28] was adopted to develop and validate the tool.

### Step 1: definition of the collaborative practice construct

A narrative review of the literature was undertaken to identify existing models, dimensions, and interprofessional collaboration measurement tools. The search strategy included the keywords “community pharmacists,” “physicians,” “collaboration,” “models,” and “interprofessional relationships.” Content analysis was performed as proposed by Mayring et al. [29] (Supplementary material 1). This qualitative deductive methodology is recommended when researchers have prior knowledge and understanding of the topic [30]. Items of Professional Collaborative Practice were extracted from questionnaires found in the literature and classified (Supplementary material 2).

### Step 2: generation of an item pool

From step 1, a pool of 156 items was generated (AISM) (Supplementary material 2). Ninety-nine of these items described similar concepts (AISM). A further 44 items were removed (AISM and VGC) because they were not related to community pharmacists’ activities or were not evaluable using Likert scales. Thirteen items were reformulated through translation to the Spanish language maintaining the original concept of the item. (AISM and VGC) The investigators developed through expert opinions of the research team (AISM, VGC and MAG) 27 new items reflecting the theoretical basis of the dimensions in the models identified from the literature, resulting in a final pool of 40 items (Supplementary material 3).

### Step 3: establishing the measurement format

A seven-point Likert scale was selected as the response format (where 1 = “never,” 2 = “very rarely,” 3 = “rarely” 4 = “occasionally,” 5 = “frequently,” 6 = “very frequently,” 7 = “always”). This seven-point scale has been proposed to be more reliable than the 5-point scale [31].

### Step 4 and 5: expert revision of the initial item pool and inclusion of validated items

Step 4 and 5 were combined as the pool of 40 items was tested in a convenient sample of 14 pharmacists to assess the ease of response, interpretability, and relevance. Slight modifications were made. The convenience sample was made of community pharmacists, independent from the researchers and based in the province of Badajoz. Pharmacists from this province did not participate any further in the study.

### Step 6: administration of the items to a development sample

There are two types of recommendations for the sample size calculation in instrument validation. Price et al. [32] recommends a minimum sample size of 200, while Bryant and Yarnold [33] is based on the subject-to-variable ratio (P), which should be no lower than 5. In our case, a sample size of 200 satisfied both recommendations.

Community pharmacists in the Spanish provinces of A Coruña, Albacete, Ciudad Real, Córdoba, Guipúzcoa, Granada, Guadalajara, Huelva, Las Palmas, Santa Cruz de Tenerife and Valencia participated in the study during 12 months. Within each province, the questionnaire was sent to all pharmacists who were participating in a study involving the provision of MRF (*n* = 110) [27]. A random sample of pharmacists who were providing usual care (i.e., not providing MRF) (*n* = 226) were also invited to participate in the study. In the random sample, participants who had not responded within a week were followed up with two phone calls. The completed questionnaires were collected during an onsite visit by members of the research team. These two samples were used in an attempt to ensure that we had a range of collaborative practices. The sample size for the usual care group was doubled as we estimated a 50% response rate.

### Step 7 and 8: content validity, reliability analysis, and optimization of the tool

#### Preliminary analysis

Missing data were imputed using decision trees (CART algorithm) [34] with the rest of the scale variables as predictors for a given item. Mahalanobis distance was calculated for all individuals from the questionnaire items. Items whose distance was greater than quartile (1–0.001) ^ (1/40) were discarded using Sidak’s correction with alpha = 0.001 from the Chi-square Test. This procedure selected three individuals as potential outliers. A further six individuals were removed as potential outliers by the PCA method according to their values in the first two components, which lead to a final sample size *n* = 276. A multidimensional scaling (MDS) was undertaken with correlations between 40 items. MDS aims to rebuild the distance matrix which, in this case, has been obtained from the correlation matrix of the original 40-item scale, using eigenvalue decomposition techniques. It is intended to be exploratory rather than inferential, and it has been used in this study for discarding items which were poorly correlated with the rest or exhibited an anomalous behaviour. Factor Analysis assumes that each variable (item) can be formulated as the linear combination of some given factors, whose value may depend on the rotation and can be correlated between themselves. The input dataset in the case of Factor Analysis is the matrix of 24 items that were finally selected, instead of a distance matrix. For these reasons, we do not expect the obtained dimensions in each method to be closely related to each other. Two dimensions were obtained with a goodness-of-fit of 63.95%). A number of items were poorly correlated (items 1–5,7-11,13,15,18,33,39,40) and were eliminated. Twenty-four items were used to validate the questionnaire**.**

#### Exploratory factor analysis – maximum likelihood method

The resultant sample of 276 pharmacists was divided into two equal groups by simple random sampling without replacement following the method by Fabrigar et al. [35]. This was undertaken to perform the CFA on the second group of data as per the method suggested by Fabrigar et al. [35] and Everett et al. [36]. In the first sample, Exploratory Factor Analysis (EFA) was applied to the 24 items using the maximum likelihood method and promax rotation. The analysis of the structure of the correlation matrix followed the Kaiser-Meyer-Olkin (KMO) measure of sampling adequacy and Bartlett’s Test of Sphericity. Individual items were evaluated for inclusion or exclusion based on factor loadings (> 0.5). Examination of the eigenvalues and scree plotting of the values determined the number of latent factors. Factor analysis with promax rotation was used. This analysis was repeated, eliminating each time the item with less load on its factor. A further 10 items were eliminated (items 6,17,21,22,26,27,29,30,32,37) based on factor loading of (< 0.5) leaving 14 items. Finally, a scree plot was used to assist in determining the number of factors present (Supplementary material 4).

#### Confirmatory factor analysis – maximum likelihood method

Using the same characteristics found in the EFA, Confirmatory Factor Analysis (CFA) was used to evaluate the degree to which the factors identified, a priori, were capable of representing the information of the correlation matrix. This evaluation was performed via several goodness-of-fit measures: standardized root mean square residual (SRMR), goodness-of-fit index (GFI), Tucker-Lewis index (TLI), and root mean square error of approximation (RMSEA).

#### Internal reliability and validity

The internal reliability was evaluated using Cronbach alpha coefficient (α > 0.7), as stated by Kelley TL et al. [37]. Empirical studies considering an N equal to or greater than 100 showed that correlations greater than or equal to 0.35 are statistically significant at a 99% confidence level [38]. The unweighted and weighted sum of scores, using factor loading of each item, was considered, and the Spearman’s correlation coefficient between the weighted and the unweighted sums was calculated to assess whether it made a difference to use one or another as the total scale. The discriminant validity of the scale was measured by using the contrasting hypothesis of difference in means of the lowest 27% and highest 27% of each of the 14 items used in the scales. Convergent validity was assessed with the Spearman’s correlation coefficient between the total sum of scores and a question that measured the self-reported degree of cooperation with the physician for each participant in the survey. The hypothesis of nullity of the correlation was tested using Spearman’s rho test. All data analyses were conducted using the statistical software R 3.5.1. (R Core Team, 2018) [39].

All the study was undertaken in accordance to guidelines of national/international/institutional or Declaration of Helsinki. Ethical approval (approval number 13/C-11) was provided by the Ethics and Research Committee of the Virgen de las Nieves University Hospital in Granada, Spain. A written information sheet was provided, and informed consent was obtained from all participants.

## Results

Three-hundred and thirty-six pharmacists were invited to participate in the study, with 285 respondents (overall response rate was 84.8%). Two hundred and twenty-six pharmacists provided usual care, of whom 177 responded (response rate of 78.3%). One hundred and eight of the 110 pharmacists involved in the MRF group responded (response rate 98.2%). The list with the 40 original items presented in the questionnaire, including their means and standard deviations obtained from the dataset after CART imputation can be found in supplementary material 5.

### Exploratory factor analysis (EFA)– maximum likelihood

**Fig. 1:**
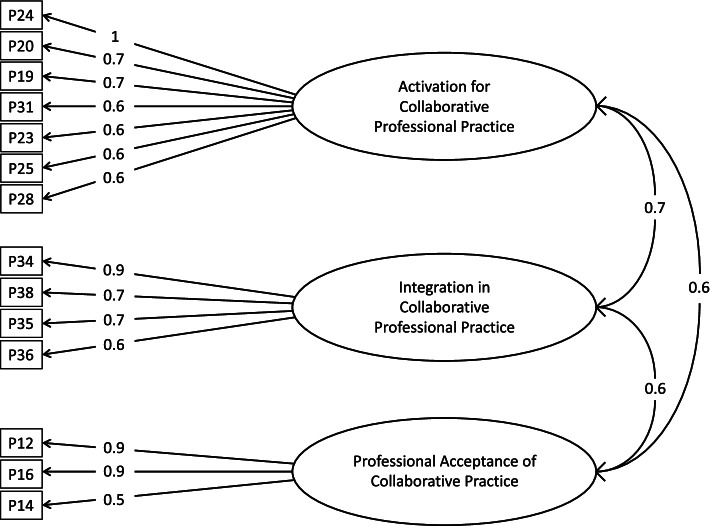
Exploratory factor analysis

Examination of the eigenvalues and scree plotting of the values determined the number of latent factors. Factor analysis with promax rotation was used. This analysis was repeated, eliminating each time the item with less load on its factor. The process ended with all items having a load on their factors greater than 0.5. A total of 10 items were deleted, resulting in a scale with 14 items and three factors (Fig. 1). The EFA suggested a structure of three factors that presents Tucker-Lewis Index = 0.945, acceptable by normal standards [40] RMSR = 0.04 (below 1/138^0.5, a value around which RMSR in acceptable scales should by Kelley, 1935) and RMSEA = 0.074 (90% confidence interval: [0.041–0.094]), which is considered acceptable. The variance explained by this structure was 62%; Factor 1 explained 27%, Factor 2 explained 20%, and Factor 3 explained 15%. The level of correlation between the factors indicated 0.74 (Factor 1 and 2), 0.60 (Factor 1 and 3), and 0.61 (Factor 2 and 3), confirming its oblique structure. Factor 1 was labeled “Activation for collaborative professional practice” and contained the seven items related to the pharmacists as the main instigator of the collaboration. Factor 2 was labeled “Integration of collaborative professional practice” and contained four items related to reciprocal interactions between both professionals. Factor 3 was labeled “Professional acceptance of collaborative practice” and contained three items related to the physician’s acceptance of the role of community pharmacists.

### Confirmatory factor analysis (CFA) – maximum likelihood method

CFA was carried out with the structure derived from the EFA and revealed three dependent factors for the reduced model of 14 items. The values obtained were SRMR = 0.056, (below 1/138^0.5, a value which RMSR in acceptable scales should be) [37], GFI = 0.889 (score which indicates fair appropriate goodness of fit) [41, 42], TLI = 0.943 (which confirms acceptability [41, 42], and RMSEA = 0.069 with confidence interval of 90% of (0.046–0.09). Taking into account the limits for acceptance, RMSEA (of 0 to 0.05 good of 0.05 and 0.08) established Schreiber et al. [43] so it can be assumed that the factorial structure is acceptable.

### Reliability and validity of the questionnaire

The Cronbach’s alpha coefficient for each factor is shown in Table 1 and the correlations items-total (Table 2). The unweighted sum of item scores was finally used as the total scale, given that the correlation between the unweighted and the weighted sum showed an almost perfect dependence (Spearman’s rho = 0.998). Cronbach’s Alpha was greater than 0.7 for each factor, and item-total correlations had values greater than 0.35 for each item. The linear correlation between the item and the scale are provided in Table 3. This correlation is known as the Homogeneity index and is normally acceptable if the correlation is above 0.35.

**Table 1:** Descriptive statistics and reliabilities for the Tool of Professional Collaborative Practice Tool

Factor	Number of items	Mean	SD	Mean -item^a^	Cronbach Alpha
**Factor 1: Activation for Professional Collaborative Practice**	7	16.4	8.6	2.3	0.902
**Factor 2: Integration in Professional Collaborative Practice**	4	8.4	4.7	2.1	0.813
**Factor 3: Professional acceptance of collaborative practice**	3	10.6	4.86	3.5	0.827

^a^Mean-item: Mean score per item of each factor. Calculated by dividing the mean of the factor by the number of items in the factor

**Table 2:** Item-total correlations without considering the corresponding item

Item 12	Item 14	Item 16	Item 19	Item 20
0.600	0.595	0.575	0.713	0.643
Item 23	Item 24	Item 25	Item 28	Item 31
0.669	0.674	0.670	0.781	0.775
Item 34	Item 35	Item 36	Item 38	
0.638	0.654	0.607	0.698

**Table 3:** Pattern matrix of the exploratory factor analysis, including factors, items, and factor loadings

Item \ Factor	Factor 1: Activation for Professional Collaborative Practice	Factor 2: Integration in Professional Collaborative Practice	Factor 3: Professional acceptance of collaborative practice
Item 12	0.03	−0.04	**0.90**
Item 14	0.13	0.11	**0.55**
Item 16	−0.09	0.04	**0.89**
Item 19	**0.69**	0.06	0.09
Item 20	**0.69**	0.15	−0.17
Item 23	**0.62**	−0.01	0.14
Item 24	**0.98**	−0.30	0.05
Item 25	**0.60**	0.16	0.01
Item 28	**0.60**	0.30	−0.01
Item 31	**0.64**	0.30	−0.03
Item 34	−0.13	**0.86**	0.01
Item 35	0.13	**0.67**	−0.08
Item 36	−0.02	**0.64**	0.12
Item 38	0.08	**0.71**	0.07

Factor loadings in bold are those considered for the construction of the factor structure

The results of the discriminant validity of the scale (summary Table 4) indicated that in all cases, the null hypothesis of equal means between upper 27% and lower 27% of individuals according to their value in a given item could be rejected, and it proved that they were different in each population and therefore discriminated between individuals.

**Table 4:** Summary of results for the discriminant validity analysis, including: mean and standard deviation of the 27% of individuals with the highest and the lowest value in each item, t statistic, degrees of freedom and *p*-value of the t-test of difference of the means for independent samples assuming unequal variances

Item	Upper 27%		Lower 27%		t	Degrees of freedom	*p*-value^1^
	Mean	SD	Mean	SD			
Item 12	6.547	0.501	1.892	0.769	43.723	125.327	9.63E-78
Item 14	4.813	0.996	1	0	33.162	74	3.69E-46
Item 16	6.187	0.586	1.378	0.488	54.456	143.028	1.62E-97
Item 19	4.747	0.871	1	0	37.237	74	1.12E-49
Item 20	3.307	1.470	1	0	13.588	74	8.50E-22
Item 23	4.387	0.852	1	0	34.402	74	2.87E-47
Item 24	4.88	0.821	1	0	40.911	74	1.46E-52
Item 25	5.133	0.935	1	0	38.292	74	1.57E-50
Item 28	4.067	1.044	1	0	25.437	74	2.55E-38
Item 31	4.507	1.107	1	0	27.424	74	1.65E-40
Item 34	4.227	1.021	1	0	27.368	74	1.89E-40
Item 35	4.04	1.096	1	0	24.022	74	1.12E-36
Item 36	4.827	1.005	1	0	32.974	74	5.48E-46
Item 38	3.36	1.158	1	0	17.645	74	3.31E-28

^1^t-test of difference of the means for independent samples assuming unequal variances

Finally, regarding convergent validity, the correlation between the Spearman’s correlation coefficient between the total sum of scores and a question that measured the self-reported degree of cooperation with the physician for each participant in the survey was 0.6824, and the results showed strong evidence that the population correlation between both factors is nonzero (Spearman’s rho test: S = 1,076,900, *p* = 9.19*10^−39^), proving that there is a convergence between the self-reported cooperation degree and the sum of the scores of the scale.

## Discussion

The study, undertaken in Spain, provided results on the development and validation of a tool to measure collaborative practice between physicians and community pharmacists from the perspective of community pharmacists. The development of this tool is critical for measuring multidisciplinary collaboration between both health care professionals, which can facilitate the optimization of healthcare systems. Both the EFA and CFA revealed three factors and confirmed the validity of the 14-item tool “Professional Collaborative Practice Tool” (Supplementary material). The first factor explaining 27% of the variance was labeled “Activation for collaborative professional practice.” This factor consisted of seven items covering physician-pharmacist interactions. During these interactions, the pharmacist takes the initiative to inform the physician of the evolution of patients’ health conditions and the outcomes derived from the provision of the professional services in the pharmacy. They therefore provide the physician with an opportunity to evaluate the risks and benefits of the professional services implemented in the pharmacy and reach an agreement to integrate them as part of their Professional Collaborative Practice with community pharmacists. Active communication between the community pharmacist and the physician is key for activating a collaborative practice. This initiative can open a channel of communication surrounding the processes, expectations, and outcomes of professional pharmacy services. It could be hypothesized that the first step should be ensuring that the physician is made aware of the service processes and their potential outcomes. In other models, such as the CWR [20] and that proposed by Bradley et al. [21] similar interactions were hypothesized as crucial elements for developing the collaborative professional relationships between community pharmacists and physicians [44]. In our model, one of the items within this factor refers to reaching an agreement between both professionals for integrating services as part of their collaborative practice. Interestingly this concept does not appear in other proposed collaborative models as a key element. The second factor was labeled “Integration in collaborative professional practice” with four items explaining 20% of the variance. This factor reflects the community pharmacist perception regarding the physician response to a collaborative approach by the community pharmacist. The items include: “This physician makes recommendations to me to improve the health care of certain patients”, “I receive feedback from this physician after making clinical recommendations”, “I ask the physician for their professional experience regarding certain professional services that I provide in the pharmacy”, where the community pharmacist seeks the views of the physician regarding the professional services provided. Lastly, one of the items refers to the community pharmacist’s perception of reaching a consensus “This physician and I jointly study strategies to improve patient health care”. In the CWR model, McDonough et al. [20] described a similar concept in Stage 3 named “Professional Relationship Expansion.” They hypothesize that, as the professional relationship progresses, communication becomes more bilateral. Key exchanges characteristics of this stage include communication, norm development, performance assessment and conflict resolution. In the proposed tool, the items of the second factor evaluate the bilateral communication between both professionals.

The third factor was labeled “Professional acceptance of collaborative practice” from the pharmacists’ perspective, and contained three items explaining 15% of the variance. This factor includes the physician’s acceptance of the active role of the community pharmacist in the effectiveness and safety monitoring of medications. It reflects the interdependence between physicians and community pharmacists in sharing decisions concerning the monitoring of the patient’s pharmacological therapy. The role specification has been proposed as a predictor of physician- community pharmacist collaboration by several authors [45, 46]. This construct may be similar to the concept of shared decision-making proposed by the CWR [20].

This study provides a valid and reliable tool with which the Professional Collaborative Practice between community pharmacists and physicians can be measured, reflecting the dynamics and interactions as perceived by community pharmacists. The analysis compared two scales using the sum of the scores of the Likert scales with and without weighting based on factor loadings and found a correlation of 0.9981. The tool allows the measurement of the Professional Collaborative Practice from the community pharmacist’s perspective using a scale that provides a single overall score for the whole questionnaire and a score for each individual factor. The arithmetic sum of the scores could be used to determine the perceived level of collaboration, rank pharmacists, and measure changes in the collaboration over time. Additionally, this data may be used to measure the impact of strategies and interventions aiming at improving the collaborative practice between both healthcare professionals. These strategies are becoming increasingly common, as the implementation of more complex professional services is expanding.

Previous studies based on the CWR model explored the concept of collaboration by suggesting that the collaborative practice transitioned through stages or phases [45]. These could be assessed over a period of time tracking the movement from to phase [46]. However, we could not find whether the stages proposed by the CWR model have been validated. Based on our analysis, we reject the stages approach hypothesis to collaborative practice. It is more probable that there is a fluid, dynamic and evolving relationship with changing scores across the three factors over time. The ability to measure changes in scores of each of the three factors as well as an overall score provides a method for proposing and selecting strategies to improve collaborative professional practice. Thus, we favor a scale approach that could be used in everyday practice. This scale to measure collaborative practice can be used by future researchers and professional organizations to evaluate collaborative practice specifically for different professional pharmaceutical services. It may also be relevant to examine the benefits of the tool from the physician perspective and to test the tool in a working or educational context and in different countries.

Some limitations must be mentioned. This tool has been validated to measure the collaborative practice between community pharmacists and physicians from the pharmacists’ perspective in Spain. Its applicability and transferability to other health care system should be tested. The tool did not assess the collaborative practice between pharmacists and physicians from the physicians’ perceptions. In future research it would be useful to develop and test scales from the perspective of the physician. The use of an identical tool both by physicians and community pharmacist could be considered however there could be an issue due to the different perspectives of the relationship are viewed.

## Conclusion

This study developed and validated a scale to measure community pharmacist and physician collaboration from the pharmacist’s perspective. The results of the present study provide a tool to measure the Professional Collaborative Practice between community pharmacists and physicians. The tool can measure the perceived level of collaboration between the two healthcare providers for services such as comprehensive medication reviews, where the collaboration is of critical importance to patient outcomes. Improvements to the working relationship between community pharmacists and physicians are considered critical for advancing the provision of overall patient-centered healthcare services. Therefore, the tool can be used not only to measure the perceived level of collaborative practice but also to measure the impact of health interventions and other professional pharmacy services that require pharmacist and physician collaboration.

## Supplementary Information

**Additional file 1: Supplementary material 1.** Content analysis process.

**Additional file 2: Supplementary material 2.** Item identification and list of articles from which constructs and items were extracted.

**Additional file 3: Supplementary material 3.** Final pool of 40 items.

**Additional file 4: Supplementary material 4**. Professional Collaborative Practice Tool.

**Additional file 5: Supplementary material 5.** List of 40 original items presented in the questionnaire, including their means and standard deviations obtained from the dataset after CART imputation. Items in bold are the 24 items that were finally included in the Exploratory Factor Analysis step (the rest were excluded in the pre-processing phase).

## Data Availability

The datasets used and/or analysed during the current study are available from the corresponding author on reasonable request.

## Abbreviations

MRF: Medication Review with Follow-up
CWR: Collaborative Working Relationship Model
ATC-P: Attitudes Towards Collaboration Instrument for Pharmacists
PPCI: Physician–Pharmacist Collaboration Instrument
ATC-GP: Attitudes Towards Collaboration Instrument for General Practitioners
MDS: Multidimensional Scaling
EFA: Exploratory Factor Analysis
CFA: Confirmatory Factor Analysis
