# Supplementary material for: Development and validation of a tool to measure collaborative practice between community pharmacists and physicians from the perspective of community pharmacists: the professional collaborative practice tool
Source: BMC Health Serv Res. 2022 May 14;22:649. doi: 10.1186/s12913-022-08027-w (PMC9107731; doi:10.1186/s12913-022-08027-w)
Supplement: Supplementary file 1 — Additional file 1: Supplementary material 1. Content analysis process. [file 12913_2022_8027_MOESM1_ESM.docx]

Supplementary material 1. Content analysis process

**Selection of the topic of analysis**

(Collaboration between CP-GP)

**Pre-Analysis**

- Literature review

- Identification of tools related to collaboration between CP-GP

**Selection of the topic of analyses**

Collaboration between CP-GP

**Development of analysis rules**

**Development of codes**

**Definition of categories**

- Professional interaction

- Exchanges characteristics

- Collaborative practice

**Definition of unit of analysis**

(According to previous tools)

**Conclusive synthesis**

Conceptual model of collaborative categories

**Definition of subcategories**

- Relationship interactions

- Trustworthiness

- Role specification

**Classification of items**

(According to the unit of analysis)
