# Supplementary material for: Development and validation of a tool to measure collaborative practice between community pharmacists and physicians from the perspective of community pharmacists: the professional collaborative practice tool
Source: BMC Health Serv Res. 2022 May 14;22:649. doi: 10.1186/s12913-022-08027-w (PMC9107731; doi:10.1186/s12913-022-08027-w)
Supplement: Supplementary file 2 — Additional file 2: Supplementary material 2. Item identification and list of articles from which constructs and items were extracted. [file 12913_2022_8027_MOESM2_ESM.docx]

Supplementary material 2. Item identification and list of articles from which constructs and items were extracted

Pharmacists-GP collaboration

(156 items)

Key elements: trust, interdependence, perceptions and expectations about the other practitioner skills, interest for collaborative practice, role definition, communication. (1)

Collaborative practice

(16 items)

Joint communication and decision-making process with the goal of satisfying the patient’s wellness and illness needs. (4,5)

Exchanges characteristics

(101 items)

These encompass the nature of social exchanges between both parties. (3)

The nature of such exchanges can range from discrete to relational

Professional interaction

(39 items)

“Interaction activities between pharmacists and physicians”. (2) Such interactions tend to be of short duration without much thought to developing a relationship or identifying new strategies to improve the patient care process.

Role specification

(33 items)

“The interdependence or complementariness of roles between health providers” (6)

Trustworthiness

(43 items)

“The trust between health providers” (6)

Relationship initiation

(25 items)

“Reflects the collaborative interests of the health provider at the beginning of the collaborative relationship” (6)

**References**

1. Bardet J.D, Vo T.H, Bedouch P, Allenet B. Physicians and community pharmacists collaboration in primary care: A review of specific models. *Research in Social & Administrative Pharmacy*. 2015;11(5):602-22.

2. McDonough R, Doucette W. Developing collaborative working relationships between pharmacists and physicians. *Journal of the American Pharmaceutical Association*. 2001;41(5):682-92.

3. Zillich A.J, McDonough R.P, Carter BL, Doucette W.R. Influential characteristics of physician/pharmacist collaborative relationships. *Annals of Pharmacotherapy*. 2004; 38(5):764-70.

4. Zillich A.J, Doucette W.R, Carter B.L, Kreiter C.D. Development and initial validation of an instrument to measure physician-pharmacist collaboration from the physician perspective. *Value in Health: International Society for Pharmacoeconomics and Outcomes Research*. 2005;8(1):59-66.

5. Van C, Mitchell B, Krass I. General practitioner–pharmacist interactions in professional pharmacy services. *Journal of Interprofessional Care*. 2011;25(5):366-72.

6. Liu Y, Doucette W.R, Farris K.B. Examining the development of pharmacist-physician collaboration over 3 months. *Research in Social & Administrative Pharmacy*. 2010;6(4):324-33.
