# Supplementary material for: Development and validation of a tool to measure collaborative practice between community pharmacists and physicians from the perspective of community pharmacists: the professional collaborative practice tool
Source: BMC Health Serv Res. 2022 May 14;22:649. doi: 10.1186/s12913-022-08027-w (PMC9107731; doi:10.1186/s12913-022-08027-w)
Supplement: Supplementary file 3 — Additional file 3: Supplementary material 3. Final pool of 40 items. [file 12913_2022_8027_MOESM3_ESM.docx]

**Supplementary material 3.** Final pool of 40 items.

**Collaborative Practice between community pharmacists and general medical practitioners from the perspective of the pharmacist**

The objective of this questionnaire is to measure the collaborative professional relationship between pharmacists and doctors. This will help to identify strategies to promote collaboration between both professionals. **We are requesting your collaboration in completing this questionnaire honestly.**

Remember: There are no right or wrong answers. All the information obtained is confidential and will be only analysed for research purposes within the programme.

| **Section one: Collaborative professional relationship**  **Consider the doctor with whom you have the most professional interactions and estimate the frequency with which the following statements occur. Please indicate the frequency level for each one by circling a number. The frequency can vary from never (1) to always (7)** | | | | | | | |
| --- | --- | --- | --- | --- | --- | --- | --- |
| **1-Never 2-Very rarely 3-Rarely 4-Occasionally 5-Frequently 6-Very frequently 7-Always** | | | | | | | |
| 1. The professional interaction with the doctor is unilateral, only initiated by me. | 1 | 2 | 3 | 4 | 5 | 6 | 7 |
| 2. I contact this doctor when I identify medication-related problems during the dispensing service. | 1 | 2 | 3 | 4 | 5 | 6 | 7 |
| 3. I initiate communications with this doctor. | 1 | 2 | 3 | 4 | 5 | 6 | 7 |
| 4. The professional interactions I have with this doctor are brief. | 1 | 2 | 3 | 4 | 5 | 6 | 7 |
| 5. The professional interactions I have with this doctor are on a one-off basis. | 1 | 2 | 3 | 4 | 5 | 6 | 7 |

| 6. I collaborate with this doctor in the managing of the pharmacological treatment of certain patients. | 1 | 2 | 3 | 4 | 5 | 6 | 7 |
| --- | --- | --- | --- | --- | --- | --- | --- |
| 7. The exchange of information I have with this doctor is minimal. | 1 | 2 | 3 | 4 | 5 | 6 | 7 |
| 8. My expectations, at a professional level, with this doctor are low. | 1 | 2 | 3 | 4 | 5 | 6 | 7 |
| 9. My role and the doctor's role in patient health care are clearly defined for both. | 1 | 2 | 3 | 4 | 5 | 6 | 7 |
| 10. This doctor is professionally credible. | 1 | 2 | 3 | 4 | 5 | 6 | 7 |
| 11. I trust the professional experience of this doctor. | 1 | 2 | 3 | 4 | 5 | 6 | 7 |
| 12. This doctor accepts that I have a role to play in the safety of the medicines prescribed. (e.g., in identifying interactions, adverse reactions, contraindications) | 1 | 2 | 3 | 4 | 5 | 6 | 7 |
| 13. I accept the role this doctor plays in patient health care. | 1 | 2 | 3 | 4 | 5 | 6 | 7 |
| 14. This doctor involves me in making-decision regarding the pharmacological treatment of certain patients. | 1 | 2 | 3 | 4 | 5 | 6 | 7 |
| 15. I trust the professional experience of this doctor in the field of medication. | 1 | 2 | 3 | 4 | 5 | 6 | 7 |
| 16. This doctor accepts that I have a role to play in the effectiveness of drug treatment. | 1 | 2 | 3 | 4 | 5 | 6 | 7 |
| 17. This doctor and I share responsibilities in decisions making about the pharmacological treatment of certain patients. | 1 | 2 | 3 | 4 | 5 | 6 | 7 |
| 18. This doctor and I agreed to have periodic face-to-face meetings to study the pharmacological treatment of certain patients. | 1 | 2 | 3 | 4 | 5 | 6 | 7 |
| 19. I inform this doctor of the changes in the health problems of patients. | 1 | 2 | 3 | 4 | 5 | 6 | 7 |
| 20. This doctor and I have reached an agreement to integrate the services that I provide in the pharmacy as part of our collaborative professional practice. | 1 | 2 | 3 | 4 | 5 | 6 | 7 |
| 21. This doctor and I openly communicate with each other. | 1 | 2 | 3 | 4 | 5 | 6 | 7 |
| 22. This doctor and I work together to establish the therapeutic objectives for the pharmacological treatment of certain patients. | 1 | 2 | 3 | 4 | 5 | 6 | 7 |

| 23. This doctor evaluates the results of the professional pharmaceutical services that I provide to certain patients. | 1 | 2 | 3 | 4 | 5 | 6 | 7 |
| --- | --- | --- | --- | --- | --- | --- | --- |
| 24. I inform this doctor of the results obtained from the services provided by the pharmacy to certain patients. | 1 | 2 | 3 | 4 | 5 | 6 | 7 |
| 25. This doctor is aware of the professional expectations I have of him/her. | 1 | 2 | 3 | 4 | 5 | 6 | 7 |
| 26. This doctor shares patient’s clinical information with me. | 1 | 2 | 3 | 4 | 5 | 6 | 7 |
| 27. I resolve disagreements with this doctor regarding how to manage the pharmacological treatment of certain patients. | 1 | 2 | 3 | 4 | 5 | 6 | 7 |
| 28. I contact this doctor so that I know his expectations about the professional services I provide at the pharmacy. | 1 | 2 | 3 | 4 | 5 | 6 | 7 |
| 29. This doctor and I have reached a collaboration agreement in our professional practice to improve patient health care. | 1 | 2 | 3 | 4 | 5 | 6 | 7 |
| 30. This doctor refers patients to the pharmacy to provide certain professional pharmaceutical services. | 1 | 2 | 3 | 4 | 5 | 6 | 7 |
| 31. I contact this doctor to know his/her expectations regarding the health of certain patients. | 1 | 2 | 3 | 4 | 5 | 6 | 7 |
| 32. I try to let this doctor know the benefits for patients of the services I provide at the pharmacy. | 1 | 2 | 3 | 4 | 5 | 6 | 7 |
| 33. Disagreements with this doctor about my role in patient health care are minimal. | 1 | 2 | 3 | 4 | 5 | 6 | 7 |
| 34. This doctor makes recommendations to me to improve the health care of certain patients. | 1 | 2 | 3 | 4 | 5 | 6 | 7 |
| 35. I ask the doctor for their professional experience regarding certain professional services that I provide in the pharmacy. | 1 | 2 | 3 | 4 | 5 | 6 | 7 |
| 36. I receive feedback from this doctor after making clinical recommendations. | 1 | 2 | 3 | 4 | 5 | 6 | 7 |
| 37. Obtaining positive results after my intervention with certain patients has contributed to improving my professional relationship with this doctor. | 1 | 2 | 3 | 4 | 5 | 6 | 7 |
| 38. This doctor and I jointly study strategies to improve patient health care. | 1 | 2 | 3 | 4 | 5 | 6 | 7 |
| 39. There is an interdependence between this doctor and me on a  professional level. | 1 | 2 | 3 | 4 | 5 | 6 | 7 |
| 40. I try to improve collaboration with this doctor by making contributions to improving the health outcomes of patients. | 1 | 2 | 3 | 4 | 5 | 6 | 7 |

| **Section two: Characteristics of the pharmacist, the doctor and the pharmacy**  **Please provide the following information** |
| --- |
| **Characteristics of the pharmacist completing the questionnaire** |
| Years of professional practice in a community pharmacy (Indicate the number of years): |
| Indicate how long you have been working in the pharmacy in which you currently carry out your professional activity (Indicate the number of years and/or months): |
| Age (Indicate your age as a number): |
| Sex (Mark with a cross):  🞎 Male  🞎 Female |
| Hours worked on average per week (Indicate the average number): |
| Type of working day (Mark with a cross):  🞎 Full day (split schedule)  🞎 Full day (intensive schedule)  🞎 Half day, morning shift  🞎 Half day, afternoon shift  🞎 Half day, morning or afternoon shift  🞎 Other (please specify): |
| Current position of the pharmacist in the pharmacy:  🞎 Pharmacy owner  🞎 Assistant pharmacist  🞎 Head pharmacist  🞎 Substitute pharmacist  🞎 Other: |
| Hours new technologies are used per day: Computer:  Mobil phone: Tablet: |
| Did you have previous experience of professional collaboration with doctors?  🞎 Yes (Specify its nature):  🞎 No |

| **Characteristics of the doctor you have considered when completing the questionnaire** |
| --- |
| Sex:  🞎 Male  🞎 Female |
| Speciality (Mark only one speciality):  🞎 General Practitioner  🞎 Other speciality (specify which) |
| Practice type (not exclusive):  🞎 Professional practice in a private entity  🞎 Professional practice in a public entity |
| Indicate your degree of collaboration with this doctor on a scale from 0 to 10, where 0 means “no collaboration” and 10 means “total collaboration” (indicate with a circle):  0 1 2 3 4 5 6 7 8 9 10 |
| **Patients shared with this doctor** |
| Number of shared patients for whom there is collaboration with this doctor: _ |
| **Characteristics of the pharmacy where you work (Mark with a cross)** |
| Pharmacy location:  🞎 Rural area  🞎 Urban area |
| Postcode: Approximate distance between the pharmacy and the doctor (in metres): |
| Pharmacy opening hours:  🞎 24 hours  🞎 12 hours  🞎 8 hours  🞎 Open only in the morning  🞎 Other |
| Number of employees in the pharmacy (indicate the number):  🞎 Pharmacy owner  🞎 Head pharmacist  🞎 Substitute pharmacist  🞎 Assistant pharmacist  🞎 Pharmacy assistant  🞎 Other |
| Pharmacy code number (first two digits of the postcode followed by the pharmacy number): |

- Please note that the original questionnaire was in Spanish. Since most of the readers would be conversant with the English language, we have undertaken a limited transcultural adaption following the methodology used by Paloma García Martín et al. Transcultural adaption and validation of the patient empowerment in long term conditions questionnaire BMC Health services research 2017, 17.324 DOI 10.1186/s12913-017-2271-7. It has not been validated but independently translated by two English native speakers and submitted to a group of experts for assessment.
