# Supplementary material for: Development and validation of a tool to measure collaborative practice between community pharmacists and physicians from the perspective of community pharmacists: the professional collaborative practice tool
Source: BMC Health Serv Res. 2022 May 14;22:649. doi: 10.1186/s12913-022-08027-w (PMC9107731; doi:10.1186/s12913-022-08027-w)
Supplement: Supplementary file 4 — Additional file 4: Supplementary material 4. Professional Collaborative Practice Tool. [file 12913_2022_8027_MOESM4_ESM.docx]

**Supplementary material 4**. Professional Collaborative Practice Tool

Collaborative practice between community pharmacist and physician from the perspective of the pharmacist

The objective of this questionnaire is to measure the collaborative professional relationship between pharmacists and physicians. This will help identify strategies to promote collaboration between both professionals. **We are requesting your collaboration in completing this questionnaire honestly.**

Remember: There are no right or wrong answers. All the information obtained is confidential

| **Consider the physician with whom you have the most professional interactions and estimate the frequency with which the following statements occur. Please indicate the frequency level for each one by circling a number. The frequency can vary from never (1) to always (7).** |
| --- |

| **1-Never 2-Very rarely 3-Rarely 4-Occasionally 5-Frequently 6-Very frequently 7-Always** | | | | |  | | |
| --- | --- | --- | --- | --- | --- | --- | --- |
| This physician evaluates the results of the professional pharmaceutical services that I provide to certain patients. | 1 | 2 | 3 | 4 | 5 | 6 | 7 |
| I contact this physician to know his/her expectations regarding the health of certain patients. | 1 | 2 | 3 | 4 | 5 | 6 | 7 |
| I inform this physician of the results obtained from the services provided by the pharmacy to certain patients. | 1 | 2 | 3 | 4 | 5 | 6 | 7 |
| This physician accepts that I have a role to play in the safety of the medicines prescribed. (e.g., in identifying interactions, adverse reactions, contraindications) | 1 | 2 | 3 | 4 | 5 | 6 | 7 |
| This physician makes recommendations to me to improve the health care of certain patients. | 1 | 2 | 3 | 4 | 5 | 6 | 7 |
| This physician accepts that I have a role to play in the effectiveness of drug treatment. | 1 | 2 | 3 | 4 | 5 | 6 | 7 |
| This physician and I jointly study strategies to improve patient health care. | 1 | 2 | 3 | 4 | 5 | 6 | 7 |
| This physician and I have reached an agreement to integrate the pharmacy´s services that I provide as part of our collaborative professional practice. | 1 | 2 | 3 | 4 | 5 | 6 | 7 |
| I ask the physician for their professional experience regarding certain professional services that I provide in the pharmacy. | 1 | 2 | 3 | 4 | 5 | 6 | 7 |
| I inform this physician of the changes in the health problems of patients. | 1 | 2 | 3 | 4 | 5 | 6 | 7 |
| This physician involves me in making decisions regarding the pharmacological treatment of certain patients. | 1 | 2 | 3 | 4 | 5 | 6 | 7 |
| I contact this physician to I know his expectations about the professional services I provide at the pharmacy. | 1 | 2 | 3 | 4 | 5 | 6 | 7 |

| I receive feedback from this physician after making clinical recommendations. | 1 | 2 | 3 | 4 | 5 | 6 | 7 |
| --- | --- | --- | --- | --- | --- | --- | --- |
| This physician is aware of the professional expectations I have of him/her. | 1 | 2 | 3 | 4 | 5 | 6 | 7 |
| Indicate your degree of collaboration with this physician on a scale from 0 to 10, where 0 means “no  collaboration” and 10 means “total collaboration”    0 1 2 3 4 5 6 7 8 9 10 | | | | | | | |
| Pharmacy code number (first two digits of the postcode followed by the pharmacy number):_____________ | | | | | | | |

- Please note that the original questionnaire was in Spanish. Since most of the readers would be conversant with the English language, we have undertaken a limited transcultural adaption following the methodology used by Paloma Garcimartin et al. Transcultural adaption and validation of the patient empowerment in the long-term conditions questionnaire BMC Health services research 2107, 17.324 DOI 10.1186/s12913-017-2271-7. It has not been validated but independently translated by two English native speakers and submitted to a group of experts for assessment.
