# Supplementary material for: Development and validation of a tool to measure collaborative practice between community pharmacists and physicians from the perspective of community pharmacists: the professional collaborative practice tool
Source: BMC Health Serv Res. 2022 May 14;22:649. doi: 10.1186/s12913-022-08027-w (PMC9107731; doi:10.1186/s12913-022-08027-w)
Supplement: Supplementary file 5 — Additional file 5: Supplementary material 5. List of 40 original items presented in the questionnaire, including their means and standard deviations obtained from the dataset after CART imputation. Items in bold are the 24 items that were finally included in the Exploratory Factor Analysis step (the rest were excluded in the pre-processing phase). [file 12913_2022_8027_MOESM5_ESM.docx]

| Item | Mean | SD |
| --- | --- | --- |
| 1 | 3.70* | 2.05 |
| 2 | 4.69 | 1.87 |
| 3 | 2.81* | 1.64 |
| 4 | 2.62* | 1.61 |
| 5 | 2.49* | 1.70 |
| **6** | **3.06** | **1.87** |
| 7 | 3.35* | 1.92 |
| 8 | 3.69* | 1.97 |
| 9 | 5.36 | 1.63 |
| 10 | 5.64 | 1.19 |
| 11 | 5.60 | 1.27 |
| **12** | **4.36** | **1.92** |
| 13 | 5.89 | 1.20 |
| **14** | **2.50** | **1.74** |
| 15 | 5.03 | 1.41 |
| **16** | **3.79** | **1.97** |
| **17** | **2.42** | **1.67** |
| 18 | 1.39 | 0.96 |
| **19** | **2.57** | **1.65** |
| **20** | **1.75** | **1.39** |
| **21** | **3.32** | **2.13** |
| **22** | **1.83** | **1.26** |
| **23** | **2.38** | **1.52** |
| **24** | **2.67** | **1.69** |
| **25** | **2.81** | **1.80** |
| **26** | **2.15** | **1.63** |
| **27** | **2.29** | **1.56** |
| **28** | **2.11** | **1.49** |
| **29** | **2.04** | **1.64** |
| **30** | **2.58** | **1.77** |
| **31** | **2.38** | **1.64** |
| **32** | **3.15** | **1.83** |
| 33 | 3.98 | 2.14 |
| **34** | **2.22** | **1.52** |
| **35** | **2.05** | **1.49** |
| **36** | **2.42** | **1.76** |
| **37** | **3.27** | **1.92** |
| **38** | **1.80** | **1.29** |
| 39 | 2.35 | 1.84 |
| 40 | 3.62 | 1.85 |

**Supplementary material 5.** List of 40 original items presented in the questionnaire, including their means and standard deviations obtained from the dataset after CART imputation. Items in bold are the 24 items that were finally included in the Exploratory Factor Analysis step (the rest were excluded in the pre-processing phase).

**Items in bold are those that made it to the final selection of 24 items**
*These items were inverted using the formula Item* = (8 – Item) in order to keep the same direction across the 40 items of the scale. The value of the mean shown here is the mean of the inverted item.
